# Synthesis of PPAR-γ Activators Inspired by the Marine Natural Product, Paecilocin A

**DOI:** 10.3390/md12020926

**Published:** 2014-02-13

**Authors:** Bin Xiao, Mingzhi Su, Eun La Kim, Jongki Hong, Hae Young Chung, Hyung Sik Kim, Jun Yin, Jee H. Jung

**Affiliations:** 1 College of Pharmacy, Pusan National University, Busan 609-735, Korea; E-Mails: windybin@hotmail.com (B.X.); sumingzhi0310@gmail.com (M.S.); eunlakim@gmail.com (E.L.K.); hyjung@pusan.ac.kr (H.Y.C.); 2 College of Traditional Chinese Materia Medica, Shenyang Pharmaceutical University, Shenyang 110016, China; E-Mail: Yinjun2002@yahoo.com; 3 College of Pharmacy, Kyung Hee University, Seoul 130-701, Korea; E-Mail: jhong@khu.ac.kr; 4 College of Pharmacy, Sungkyunkwan University, Suwon 440-746, Korea; E-Mail: hkims@skky.edu

**Keywords:** PPAR-γ, diabetes, phthalimide, luciferase assay, docking simulation, cell proliferation

## Abstract

A series of *N*-substituted phthalimide derivatives were synthesized based on a pharmacophore study of paecilocin A (a natural PPAR-γ agonist) and synthetic leads. The introduction of hydrophilic and hydrophobic groups to the phthalimide skeleton yielded compounds **3**–**14**. Compound **7** showed significant PPAR-γ activation in a luciferase assay using rat liver Ac2F cells. Docking simulations showed that a free hydroxyl group on the phthalimide head and a suitable hydrophilic tail, including a phenyl linker, were beneficial for PPAR-γ activation. Compound **7** and rosiglitazone concentration-dependently activated PPAR-γ with EC_50_ values of 0.67 μM and 0.028 μM, respectively. These phthalimide derivatives could be further investigated as a new class of PPAR-γ ligands.

## 1. Introduction

Peroxisome proliferator-activated receptors (PPARs) are members of the nuclear receptor superfamily of ligand-activated transcription factors and comprise three isoforms, that is, PPAR-α, -β/δ and -γ [1,2,3,4]. PPAR-γ is predominantly expressed in adipose tissue, macrophages, monocytes, intestinal cells, skeletal muscle and endothelium and plays an important role in the regulation of insulin sensitivity, lipid metabolism, adipogenesis and glucose homeostasis [5]. On the other hand, PPAR-γ agonists, such as thiazolidinediones (TZDs, such as rosiglitazone and troglitazone), are used clinically to treat type II diabetes mellitus, to lower blood glucose levels and to improve insulin sensitivity [6]. In addition, a series of l-tyrosine analogues (e.g., farglitazar and muraglitazar) have been developed as PPAR-γ agonists and subjected to phase II clinical trials [7,8,9], whereas linoleic acid, α-linolenic acid and prostanoid 15-deoxy-Δ^12,14^-PGJ_2_ are putative endogenous ligands for PPAR-γ, with relatively low affinities [10,11].

**Figure 1:**
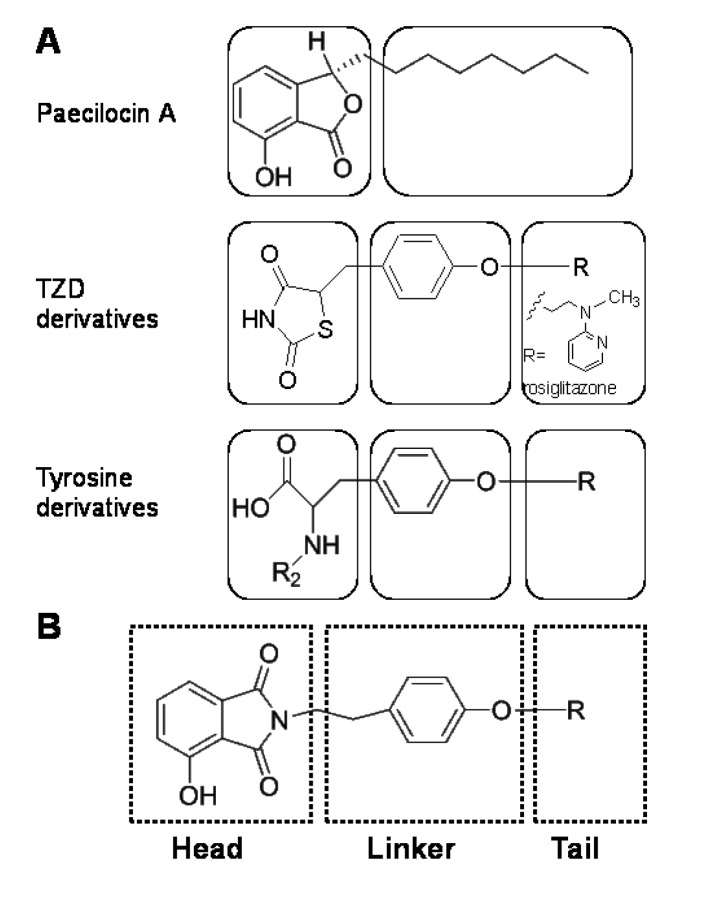
(**A**) Simplified topologies of paecilocin A and typical synthetic PPAR-γ agonists. Paecilocin A contains a hydrophilic 3-hydroxy phthalide moiety and a hydrophobic octyl chain; both thiazolidinedione (TZD) and tyrosine derivatives employ a phenol moiety as a linker between their hydrophilic heads and hydrophobic tails. (**B**) The *N*-substituted phthalimide skeleton of PPAR-γ agonists; a 3-hydroxy phthalimide moiety acts as the head, a phenol moiety as the central linker and a hydrophobic or hydrophilic substituent as the tail.

Endogenous PPAR-γ ligands typically contain a free carboxylic acid head and an unsaturated alkyl chain terminus (the tail), whereas TZDs and l-tyrosine analogues have carbonyl, amino or carboxyl groups at their hydrophilic heads and a phenol moiety, which acts as a linker between the head and tail. Hydrophilic head groups form H-bonds with key amino acid residues (Tyr^473^, His^449^, His^323^ and Ser^289^) of the ligand-binding domain (LBD) of PPAR-γ and stabilize PPAR-γ in the conformation required for successful co-activator recruitment [12,13,14,15]. In our previous study, we isolated paecilocin A (Figure 1), a new type of PPAR-γ agonist, from the jellyfish-derived fungus, *Paecilomyces* *variotii* [16], and based on the results of a pharmacophore study on paecilocin A and rosiglitazone, we proposed that the 3-hydroxy phthalide moiety of paecilocin A functions as a hydrophilic head and forms H-bonds with the key amino acid residues of the LBD of PPAR-γ [13].

The easily accessible phthalimide moiety has often been employed as a pharmacophore in drug development [17], and some phenethylphenylphthalimide derivatives have been reported to be PPAR-γ agonists. However, the phthalimide moiety of these derivatives does not contain a free hydroxyl group, and it was speculated to serve as a hydrophobic tail, which settles in the hydrophobic region of the PPAR-γ LBD [18]. On the contrary, the phthalide moiety of paecilocin A was speculated to behave as a polar head group that forms H-bonds with key amino acid residues in the hydrophilic pocket of PPAR-γ LBD (Figure 1A) [13]. Considering the structural similarity between the phthalimide moiety and the phthalide moiety of paecilocin A, we postulated the introduction of hydroxyl groups would allow the phthalimide moiety to be utilized as a polar head group and that this modification could be further extended using linker and tail groups to generate potential PPAR-γ ligands (Figure 1B). In the present study, phthalimide-derived molecules were designed, produced and evaluated with respect to PPAR-γ activation in rat liver Ac2F cells.

## 2. Results and Discussion

### 2.1. Chemistry

**Scheme 1:**
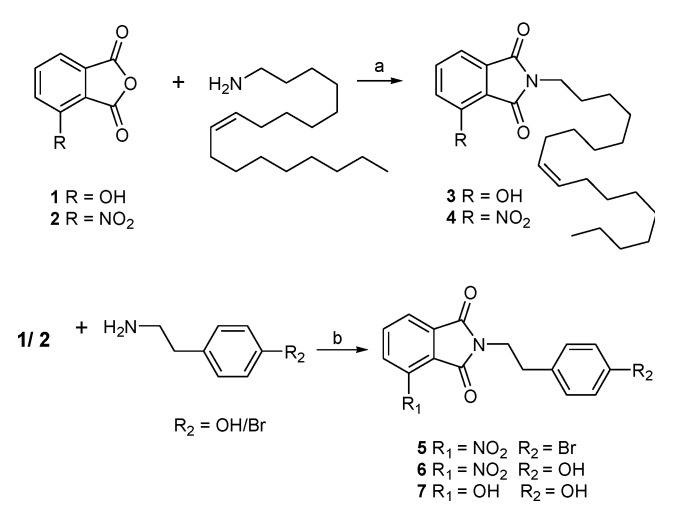
Synthesis of phthalimide derivatives (**3**–**7**, yield: ~90%). Reagents and conditions: (**a**) CH_3_COOH, 85 °C, 14 h; (**b**) CH_3_COOH, 85 °C, overnight.

*N*-substituted phthalimides can be prepared by heating phthalic anhydride with various *N*-containing reagents [19]. In the present study, this was performed by treating phthalic anhydrides (**1**, **2**) with various amines in the presence of acetic acid to generate corresponding *N*-substituted phthalimides (Scheme 1). Compounds **3** and **4** were synthesized to imitate the skeletons of lipidic PPAR-γ agonists and paecilocin A by treating phthalic anhydrides with oleylamine, whereas compounds **5**–**7** were synthesized to imitate the topology of synthetic PPAR-γ agonists (e.g., rosiglitazone and farglitazar) by connecting the phthalimide head with p-hydroxy- or p-bromo-phenethyl groups.

**Scheme 2:**
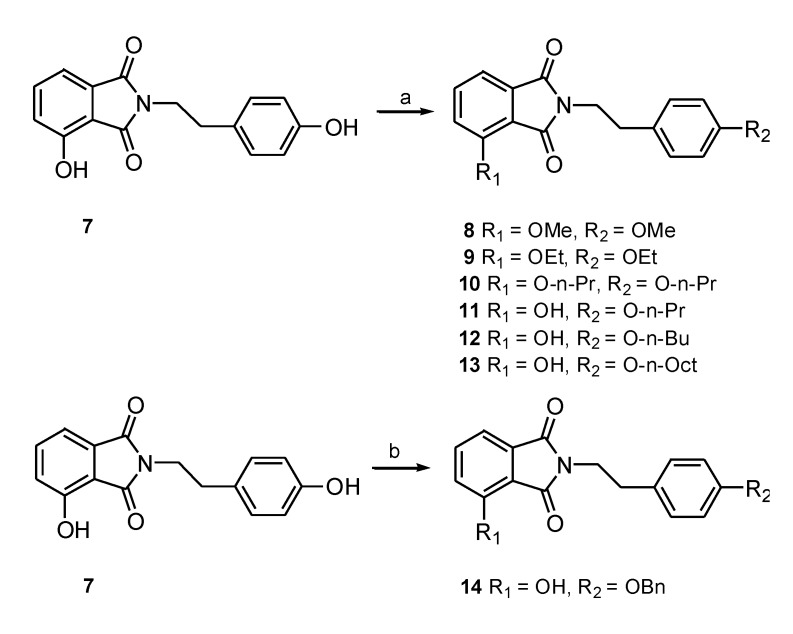
Synthesis of phthalimide derivatives (**8**–**10**, yield: ~25%; **11** and **12**, yield: ~75%; **13**, yield: ~60%; **14**, yield: ~60%). Reagents and conditions: (**a**) RI (MeI, EtI, n-PrI, n-BuI, n-OctI) Ag_2_O, stir, CH_3_CN, reflux for 12 h; (**b**) benzyl chloride, K_2_CO_3_, NaI, DMF, stir, RT for 4 h.

Previous studies have shown that a large hydrophobic substituent, such as a hetero-aromatic group, provides a good tail for PPAR-γ agonists [20]. Accordingly, we speculated that connecting the phenoxyl moiety with a hydrophobic tail designed to occupy the hydrophobic binding pocket in the LBD of PPAR-γ might enhance binding affinity. Therefore, compound **7** was derivatized by treating it with iodomethane, iodoethane, iodopropane, iodobutane, iodooctane or benzyl chloride to produce the hydroxy-substituted analogues **8**–**14** (Scheme 2). The major product obtained was the result of the monoalkylation of the hydroxyl group on the phenethyl moiety (**11**), and the dialkylated product (**10**) was produced at low levels. The monobenzylated derivative (**14**) was also prepared.

### 2.2. Biological Activity

**Figure 2:**
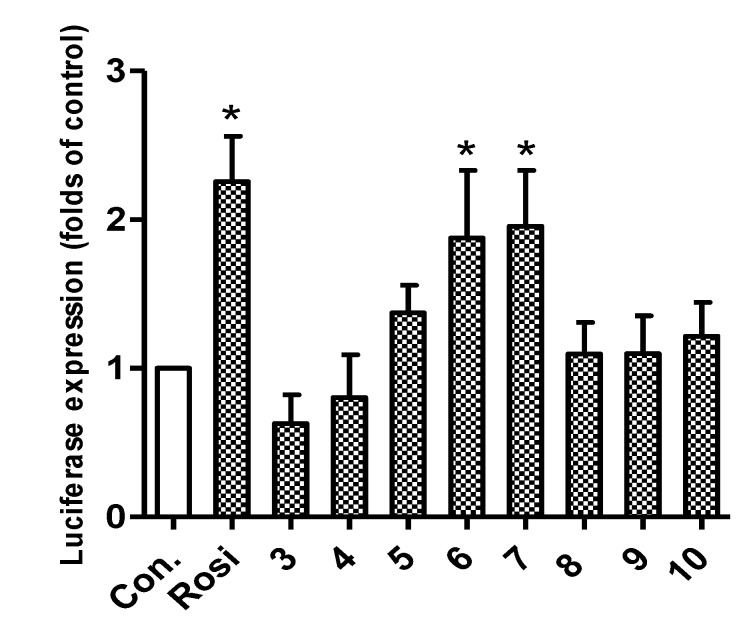
*In vitro* assay of PPAR-γ activation by phthalimides **3**–**10** and by rosiglitazone at 10 μM in rat liver Ac2F cells. Con., the negative control, transfected with a plasmid containing PPAR response element (PPRE) and pcDNA3; Rosi (rosiglitazone) was used as the positive reference control. Treated cells were transiently transfected with PPRE plus full-length human PPAR-γ1 expression vector (pFlag)-PPAR-γ1. Luciferase expressions (folds of the control) are the means ± SDs (*n* = 3). * *p* < 0.05.

**Figure 3:**
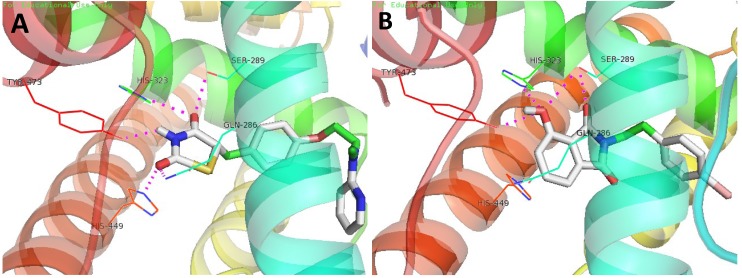
The 3D putative binding modes of rosiglitazone or **7** with the ligand-binding domain (LBD) of PPAR-γ. (**A**) Rosiglitazone interacts with the key amino acid residues, Tyr^473^, His^449^, His^323^, Ser^289^ and Glu^286^, in the PPAR-γ binding pocket (−8.2 kcal/mol). (**B**) The binding mode of **7** (−8.4 kcal/mol), which interacts with the key amino acid residues, Tyr^473^, His^323^ and Ser^289^, in the PPAR-γ binding pocket.

Synthesized compounds were subsequently evaluated for PPAR-γ activation using a luciferase assay in Ac2F cells (a rat liver cell line). The potencies of compounds **6** and **7** were comparable to rosiglitazone at 10 μM (Figure 2). In docking simulations [21], the hydrophilic head of compound **7** was found to form H-bonds with the key amino acid residues (Tyr^473^, His^323^ and Ser^289^) of the LBD of PPAR-γ (Figure 3), in the same manner as rosiglitazone [13]. Compounds **3**, **4** and **6**–**14** also appeared to bind to the LBD of PPAR-γ and to interact with key amino acid residues (see Appendix Table A1). Alkylated members (**3** and **4**) showed low binding affinities, whereas **6** and **7** showed high binding affinities, which suggested that the aromatic linker provided better binding than a long alkyl chain, possibly because the alkyl chain is too bulky to fit into the binding pocket. Compounds **3** and **4** produced even lower levels of PPAR-γ activation than the control, possibly because of their cytotoxicities.

**Figure 4:**
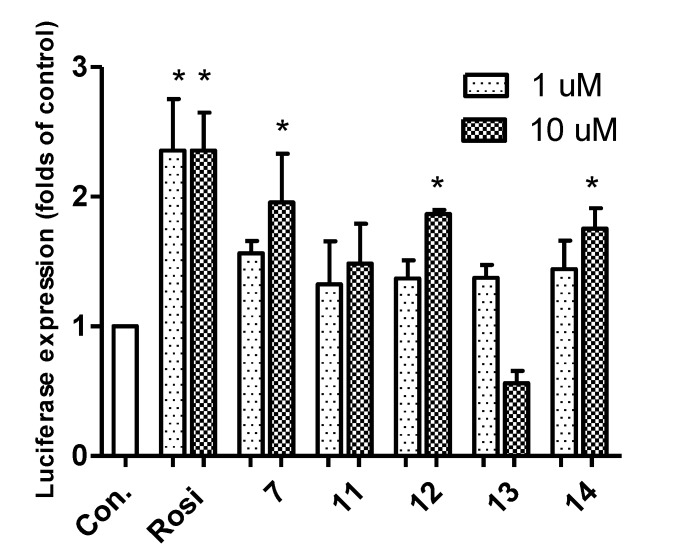
*In vitro* assay of PPAR-γ activation by phthalimides **7**, **11**–**14** and by rosiglitazone at 1 μM or 10 μM in rat liver Ac2F cells. Con., the negative control, transfected with plasmid containing PPRE and pcDNA3. Rosi, rosiglitazone. Rosiglitazone was used as the positive reference control to monitor the activation of the luciferase reporter. Compound-treated cells were transiently transfected with PPRE plus pFlag-PPAR-γ1. Luciferase expressions (folds of the control) are the means ± SDs (*n* = 3). * *p* < 0.05.

**Figure 5:**
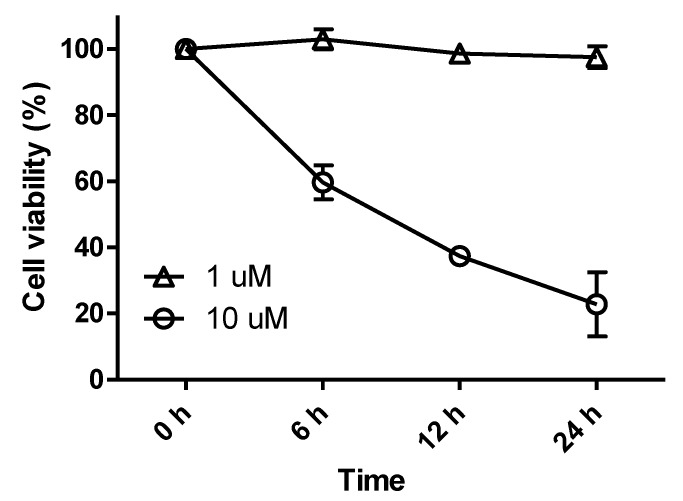
Cell viabilities of rat liver Ac2F cells treated with compound **13**. Cells were treated with compound **13** for 24 h at concentrations of 1 μM or 10 μM. Cell proliferation ratios are the means ± SDs (*n* = 6).

**Figure A1:**
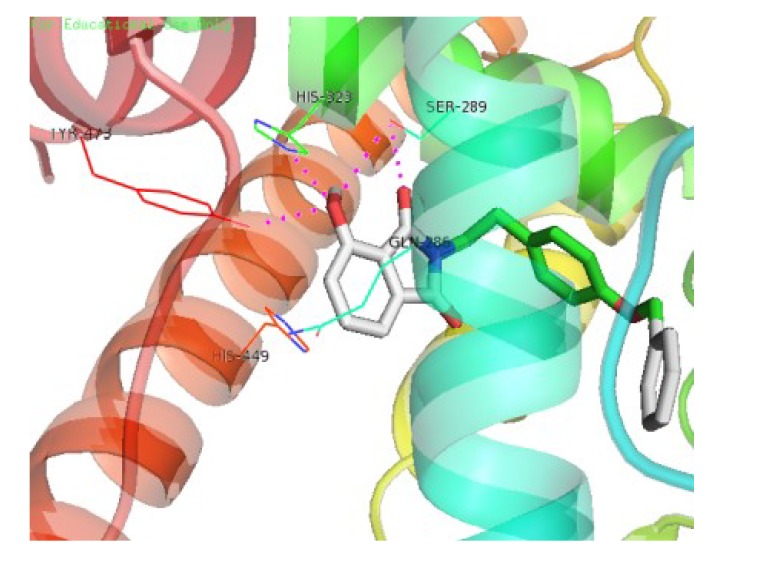
The 3D putative binding mode of **14** (−9.4 kcal/moL) with PPAR-γ LBD. Compound **14** interacts with the key amino acid residues (Tyr^473^, His^343^, and Ser^289^) in the ligand binding pocket of PPAR-γ.

Secondary *N*-substituted phthalimide derivatives (**11**–**14**) activated PPAR-γ more than the control (rosiglitazone) at 10 μM, except compound **13** (Figure 4). As was expected, secondary derivatives with a free hydroxyl group on the phthalimide moiety (**11**–**14**) were more effective than *O*-alkylated derivatives (**8**–**10**) (Figure 2). These findings indicated that the free 3-OH on the phthalimide moiety was essential for inducing PPAR-γ activity, and this notion was consistent with the strong docking affinities of **11**–**14** to the LBD of PPAR-γ (see Appendix Table A1). Interestingly, the PPAR-γ agonistic activity of a long octyl tail derivative (**13**) sharply decreased at a concentration of 10 μM, which was similar to that observed for compounds **3** and **4**, which also have a long hydrophobic tail (Figure 2 and Figure 4). These observations could be due to the cytotoxic effects of derivatives with long hydrophobic tails. Indeed, compound **13** exhibited strong cytotoxicity toward Ac2F cells at 10 μM (Figure 5). Although **7** and **14** formed similar H-bond networks in docking simulations (Figure 3B and Appendix Figure A1), compound **14**, which possess a benzyl group, seemed reluctant to locate in the binding site, based on its smaller NP (the number of modes located in the binding pocket) and AI (affinity of the top-ranked mode) values than compound **7** (see Appendix Table A1).

**Figure 6:**
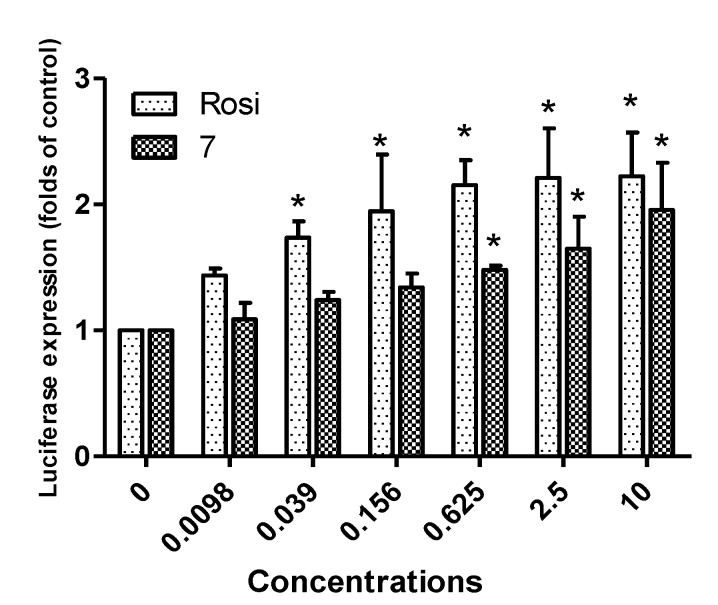
Dose-dependent PPAR-γ agonistic activities of rosiglitazone and compound **7**. Ac2F cells were stimulated with rosiglitazone or compound **7** at various concentrations (0.0098 μM~10 μM). Con., the negative control, transfected with plasmid containing PPRE and pcDNA3. Rosi (rosiglitazone) was used as the positive reference control to monitor the activation of luciferase reporter. Treated cells were transiently transfected with PPRE plus pFlag-PPAR-γ1. Luciferase expressions (fold *versus* the control) are presented as the means ± SDs (*n* = 3). * *p* < 0.05.

**Figure 7:**
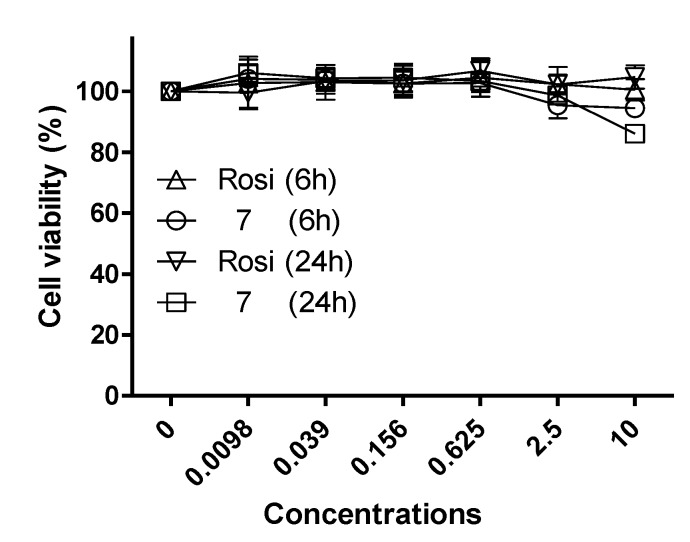
The effects of rosiglitazone and compound **7** on the viability of Ac2F cells. Cells were treated with rosiglitazone and compound **7** for 6 h or 24 h at various concentrations (0.0098 μM~10 μM). Cell proliferation ratios are presented as the means ± SDs (*n* = 6).

Based on the above-mentioned results, **7** was selected as a potential lead compound and further evaluated at different concentrations (0.0098 μM~10 μM) *versus* rosiglitazone (Figure 6). Both compound **7** and rosiglitazone exhibited concentration-dependent PPAR-γ activation. The activity of compound **7** was comparable to that of rosiglitazone at the concentration of 10 μM. Cell proliferation enhancement by compound **7** was compared with that of rosiglitazone at different concentrations (Figure 7). No significant change in cell viability was induced by either compound after 6 h of treatment at concentrations of one and 10 μM, which exclude the possibility that enhanced proliferation was responsible for the observed increase in luciferase expression. Furthermore, the low cytotoxicity of compound **7** toward Ac2F cells demonstrated its potential safety, a key factor for a lead development compound.

## 3. Experimental Section

### 3.1. Chemistry

^1^H and ^13^C NMR spectra were recorded on a Varian Unity 400 MHz NMR spectrometer, and chemical shifts are reported with respect to respective residual solvents or deuterated solvent peaks (δ_H_ 3.30 and δ_C_ 49.0 for CD_3_OD, δ_H_ 7.24 and δ_C_ 76.8 for CDCl_3_). FABMS data were obtained using a JEOL JMS SX-102A spectrometer (JEOL, Atlanta, GA, USA). HPLC was performed using a YMC ODS-H80 column (250 × 10 mm, 4 µm, 80 Å) or a C18-5E Shodex packed column (250 × 10 mm, 5 µm, 100 Å; YMC Co., Ltd, Kyoto, Japan) and a Shodex RI-71 detector (Triad Scientific, Inc., Manasquan, NJ, USA). All reagents were purchased from Sigma-Aldrich (Saint Louis, MO, USA) and used as received.

#### 3.1.1. Preparation of *N*-Substituted Phthalimides **3**–**7**

A mixture of amine (1.2 equivalent) and phthalic anhydride in aqueous glacial acetic acid (1 M) was stirred and heated under reflux overnight. Products were precipitated by adding water, filtered and washed thoroughly with water. Residues were diluted with MeOH, dried with MgSO_4_ and evaporated to provide the crude products, **3**–**7** (yield: ~90%).

3-Hydroxy-*N*-oleyl phthalimide (**3**). White powder;^ 1^H NMR (CDCl_3_, 400 MHz): δ 0.85 (t, *J* = 7.2 Hz, 3H), 1.20 (m, 22H), 1.63 (m, 2H), 1.98 (m, 4H), 3.60 (t, *J* = 7.6 Hz, 2H), 5.30 (m, 2H), 7.13 (d, *J* = 8.4 Hz, 1H), 7.34 (d, *J* = 7.6 Hz, 1H), 7.54 (t, *J* = 8.0 Hz, 1H); ^13^C NMR (CDCl_3_, 100 MHz): δ 170.3, 167.9, 154.9, 137.0, 136.6, 132.2, 131.9, 130.7, 39.0, 32.8, 32.1, 30.0, 29.9, 29.9, 29.7, 29.6, 29.5, 29.4, 29.4, 28.8, 27.4, 27.4, 27.1, 22.9, 14.3; FABMS *m/z* 414 [M + H]^+^. 

3-Nitro-*N*-oleyl phthalimide (**4**). White powder; ^1^H NMR (CDCl_3_, 400 MHz): δ 0.85 (t, *J* = 7.2 Hz, 3H), 1.20 (m, 22H), 1.63 (m, 2H), 1.98 (m, 4H), 3.60 (t, *J* = 7.6 Hz, 2H), 5.30 (m, 2H), 7.68 (m, 2H), 7.82 (m, 2H); ^13^C NMR (CDCl_3_, 100 MHz): δ 165.7, 162.9, 145.3, 136.6, 135.6, 134.2, 132.0, 130.7, 39.8, 32.8, 32.1, 30.0, 29.9, 29.9, 29.7, 29.6, 29.5, 29.4, 29.4, 28.8, 27.4, 27.4, 27.1, 22.9, 14.3; FABMS *m/z* 443 [M + H]^+^.

3-Nitro-*N*-(*p*-bromo-phenethyl) phthalimide (**5**). White powder; ^1^H NMR (CDCl_3_, 400 MHz): δ 2.95 (t, *J* = 7.2 Hz, 2H), 3.91 (m, 2H), 7.01 (d, *J* = 8.4 Hz, 2H), 7.39 (d, *J* = 8.4 Hz, 2H), 7.88 (t, *J* = 7.6 Hz, 1H), 8.08 (m, 2H); ^13^C NMR (CDCl_3_, 100 MHz): δ 165.7, 162.9, 145.3, 136.6, 135.6, 134.2, 132.0, 130.7, 128.8, 127.3, 123.9, 121.0, 39.8, 33.9; FABMS *m/z* 375 [M + H]^+^.

3-Nitro-*N*-(*p*-hydroxy-phenethyl) phthalimide (**6**). White powder; ^1^H NMR (CD_3_OD, 400 MHz): δ 2.85 (t, *J* = 7.6 Hz, 2H), 3.82 (m, 2H), 6.64 (d, *J* = 8.8 Hz, 2H), 7.00 (d, *J* = 8.8 Hz, 2H), 7.95 (t, *J* = 7.6 Hz, 1H), 8.06 (d, *J* = 7.6 Hz, 2H), 8.12 (d, *J* = 8.0 Hz, 2H); ^13^C NMR (CDCl_3_, 100 MHz): δ 165.8, 163.0, 154.7, 154.6, 135.5, 130.2, 130.1, 128.7, 127.2, 115.8, 115.7, 40.4, 33.6; FABMS *m/z* 313 [M + H]^+^.

3-Hydroxy-*N*-(*p*-hydroxy-phenethyl) phthalimide (**7**). White powder; ^1^H NMR (CD_3_OD, 400 MHz): δ 2.81 (t, *J* = 7.2 Hz, 2H), 3.74 (t, *J* = 7.6 Hz, 2H), 6.63 (d, *J* = 8.4 Hz, 2H), 6.98 (d, *J* = 8.4 Hz, 2H), 7.09 (d, *J* = 8.0 Hz, 1H), 7.27 (d, *J* = 7.2 Hz, 1H), 7.52 (t, *J* = 7.6 Hz, 1H); ^13^C NMR (CD_3_OD, 100 MHz): δ 168.4, 168.1, 155.9, 155.2, 135.8, 133.6, 129.6, 129.2, 122.9, 115.1, 115.0, 114.4, 39.1, 33.3; FABMS *m/z* 284 [M + H]^+^.

#### 3.1.2. General Procedure for the Synthesis of *N*-substituted Phthalimides **8**–**13**

To a solution of 3-hydroxy-*N*-(*p*-hydroxy-phenethyl) phthalimide **7** (13 mg, 0.046 mmoL) in CH_3_CN (1.5 mL), RI (CH_3_I: 6.0 μL, *ca*. 0.09 mmoL; CH_3_CH_2_I: 7.0 μL, *ca*. 0.09 mmoL; CH_3_(CH_2_)_2_I: 9.0 μL, *ca*. 0.09 mmoL; CH_3_(CH_2_)_3_I: 10.1 μL, *ca*. 0.09 mmoL; CH_3_(CH_2_)_7_I: 15.6 μL, *ca*. 0.09 mmoL) and Ag_2_O (10 mg, 0.04 mmoL) were added. The mixture was then heated under reflux with stirring for 12 h. Solid material was removed by filtration and the solvent by evaporation, and the solid material obtained was purified by RP-HPLC using 90% aqueous MeOH as the eluant to give **8**–**10** (yield: ~25%) or **11**–**13** (yield: ~75%, ~75% and ~60%, respectively).

3-Methoxy-*N*-(*p*-methoxy-phenethyl) phthalimide (**8**). White powder; ^1^H NMR (CDCl_3_, 400 MHz): δ 2.89 (m, 2H), 3.75 (s, 3H), 3.83 (m, 2H), 4.00 (s, 3H), 6.80 (d, *J* = 8.8 Hz, 2H), 7.16 (m, 3H), 7.39 (d, *J* = 7.2 Hz, 1H), 7.62 (t, *J* = 7.6 Hz, 1H); ^13^C NMR (CDCl_3_, 100 MHz): δ 168.2, 167.1, 158.5, 156.8, 136.2, 136.2, 134.4, 130.4, 130.0, 130.0, 117.6, 115.6, 114.1, 114.1, 56.5, 55.4, 39.5, 33.9; FABMS *m/z* 312 [M + H]^+^.

3-Ethoxy-*N*-(*p*-ethoxy-phenethyl) phthalimide (**9**). White powder; ^1^H NMR (CDCl_3_, 400 MHz): δ 1.37 (t, *J* = 7.2 Hz, 3H), 1.52 (t, *J* = 7.2 Hz, 3H), 2.87 (m, 2H), 3.81 (m, 2H), 3.97 (t, *J* = 6.8 Hz, 2H), 4.24 (t, *J* = 6.8 Hz, 2H), 6.79 (d, *J* = 8.4 Hz, 2H), 7.14 (m, 3H), 7.37 (d, *J* = 7.6 Hz, 1H), 7.59 (t, *J* = 7.6 Hz, 1H); ^13^C NMR (CDCl_3_, 100 MHz): δ 168.1, 167.1, 157.8, 156.3, 136.1, 134.6, 130.3, 130.0, 130.0, 118.7, 117.7, 115.5, 114.7, 114.7, 65.2, 63.6, 39.5, 34.0, 15.1, 14.8; FABMS *m/z* 340 [M + H]^+^.

3-Propoxy-*N*-(*p*-propoxy-phenethyl) phthalimide (**10**). White powder; ^1^H NMR (CDCl_3_, 400 MHz): δ 1.00 (t, *J* = 7.2 Hz, 3H), 1.08 (t, *J* = 7.2 Hz, 3H), 1.76 (m, 2H), 1.90 (m, 2H), 2.87 (m, 2H), 3.80 (m, 2H), 3.86 (t, *J* = 6.8 Hz, 2H), 4.11 (t, *J* = 6.8 Hz, 2H), 6.79 (d, *J* = 8.4 Hz, 2H), 7.14 (m, 3H), 7.37 (d, *J* = 7.2 Hz, 1H), 7.59 (t, *J* = 7.6 Hz, 1H); ^13^C NMR (CDCl_3_, 100 MHz): δ 168.2, 167.0, 157.5, 156.2, 136.0, 134.4, 130.4, 130.1, 130.1, 118.7, 117.6, 115.5, 115.5, 115.5, 71.0, 69.5, 39.5, 33.9, 22.8, 22.5, 10.9, 10.6; FABMS *m/z* 368 [M + H]^+^.

3-Hydroxy-*N*-(*p*-propoxy-phenethyl) phthalimide (**11**). White powder; ^1^H NMR (CDCl_3_, 400 MHz): δ 1.08 (t, *J* = 6.0 Hz, 3H), 1.90, (m, 2H), 2.89 (t, *J* = 7.2 Hz, 2H), 3.83 (t, *J* = 6.4 Hz, 2H), 4.12 (t, *J* = 6.4 Hz, 2H), 6.75 (d, *J* = 6.4 Hz, 2H), 7.09 (d, *J* = 6.4 Hz, 2H), 7.16 (d, *J* = 6.8 Hz, 1H), 7.38 (d, *J* = 6.0 Hz, 1H), 7.60 (t, *J* = 6.0 Hz, 1H); ^13^C NMR (CDCl_3_, 100 MHz): δ 168.3, 167.1, 156.5, 154.6, 136.1, 134.4, 130.4, 130.2, 130.2, 118.8, 117.6, 115.6, 115.6, 115.5, 71.0, 39.5, 33.9, 22.5, 10.6; FABMS *m/z* 326 [M + H]^+^.

3-Hydroxy-*N*-(*p*-butoxy-phenethyl) phthalimide (**12**). White powder; ^1^H NMR (CDCl_3_, 400 MHz): δ 0.97 (t, *J* = 7.2 Hz, 3H), 1.52, (m, 2H), 1.85, (m, 2H), 2.87 (t, *J* = 7.6 Hz, 2H), 3.81 (t, *J* = 7.6 Hz, 2H), 4.14 (t, *J* = 6.4 Hz, 2H), 6.72 (d, *J* = 8.4 Hz, 2H), 7.09 (d, *J* = 8.4 Hz, 2H), 7.14 (d, *J* = 8.8 Hz, 1H), 7.36 (d, *J* = 7.2 Hz, 1H), 7.58 (t, *J* = 7.2 Hz, 1H); ^13^C NMR (CDCl_3_, 100 MHz): δ 168.3, 167.2, 156.5, 154.6, 136.1, 136.1, 134.4, 130.4, 130.2, 118.8, 117.6, 115.6, 115.6, 115.4, 69.3, 39.5, 33.9, 31.2, 19.3, 14.0; FABMS *m/z* 340 [M + H]^+^.

3-Hydroxy-*N*-(*p*-octyloxy-phenethyl) phthalimide (**13**). White powder; ^1^H NMR (CDCl_3_, 400 MHz): δ 0.86 (t, *J* = 7.2 Hz, 3H), 1.27 (m, 8H), 1.48 (m, 2H), 1.87 (m, 2H), 2.87 (m, 2H), 3.80 (m, 2H), 4.12 (t, *J* = 6.8 Hz, 2H), 6.79 (d, *J* = 8.4 Hz, 2H), 7.14 (m, 3H), 7.37 (d, *J* = 7.2 Hz, 1H), 7.59 (t, *J* = 7.6 Hz, 1H); FABMS *m/z* 396 [M + H]^+^.

#### 3.1.3. General Procedure for the Synthesis of 3-Hydroxy-*N*-(*p*-benzyl-phenethyl) Phthalimide (**14**)

To a suspension of 3-hydroxy-*N*-(*p*-benzyloxy-phenethyl) phthalimide **7** (13.4 mg, 0.047 mmoL), K_2_CO_3_ (6.5 mg, 0.047 mmoL) and NaI (2.6 mg, 0.02 mmoL) in DMF (2 mL) was added benzyl chloride (6.0 μL, *ca*. 0.06 mmoL). The mixture was then stirred for 30 min at 0 °C and for 4 h at room temperature, acidified with aqueous 6 M HCl and extracted with EtOAc. The organic layer was successively washed with H_2_O and brine, dried with MgSO_4_ and evaporated to give the crude product, which was purified by RP-HPLC using 85% aqueous MeOH as the eluant to give **14** (yield 95%): white powder; ^1^H NMR (CDCl_3_, 400 MHz): δ 2.89 (t, *J* = 7.2 Hz, 2H), 3.83 (t, *J* = 7.6 Hz, 2H), 5.31 (s, 2H), 6.73 (d, *J* = 8.4 Hz, 2H), 7.11 (d, *J* = 8.4 Hz, 2H), 7.17 (d, *J* = 8.4 Hz, 1H), 7.31 (t, *J* = 7.2 Hz, 1H), 7.38 (t, *J* = 7.6 Hz, 3H), 7.47 (d, *J* = 7.6 Hz, 2H), 7.56 (t, *J* = 7.6 Hz, 1H); ^13^C NMR (CDCl_3_, 100 MHz): δ 168.1, 167.0, 155.9, 154.5, 136.1, 136.0, 134.5, 130.4, 130.2, 130.2, 129.0, 129.0, 128.4, 127.0, 127.0, 119.7, 118.2, 116.0, 115.6, 115.6, 71.1, 39.6, 33.9; FABMS *m/z* 374 [M + H]^+^.

### 3.2. Luciferase Assay

Rat liver Ac2F cells were obtained from the American Type Culture Collection (ATCC, Rockville, MD, USA). Cells were grown in Dulbecco’s Modified Eagle Medium (DMEM, Nissui, Tokyo) containing 2 mM l-glutamine, 100 mg/mL streptomycin, 2.5 mg/L amphotericin B and 10% heat-inactivated fetal bovine serum (FBS) and maintained in a humidified atmosphere containing 5% CO_2_ at 37 °C. The TK-PPRE × 3-luciferase reporter plasmid containing three copies of the PPAR response element (PPRE) in acyl CoA oxidase promoter was generously donated by Dr. Christopher K. Glass (University of California, San Diego, CA, USA). The pcDNA3 expression vector and full-length human PPAR-γ1 expression vector (pFlag-PPAR-γ1) were generously donated by Dr. Chatterjee (University of Cambridge, Addenbrooke’s Hospital, Cambridge, UK). For luciferase assays, plasmids were transfected into Ac2F cells in a 48-well plate (5 × 10^4^ cells/well) with effector plasmids and the TK-PPRE × 3-luciferase reporter plasmid (1 μg/well) plus pcDNA3(0.1 μg/well) or pFlag-PPAR-γ1 (0.1 μg/well) using Lipofectamine™ 2000 (Invitrogen Co., Carlsbad, CA, USA), according to the manufacturer’s instructions. After transfection for 4 h, conditioned media was replaced with complete medium, and cells were incubated for an additional 20 h. The medium was then removed, and cells were exposed in serum-free media to rosiglitazone or test compounds for 6 h, washed with PBS and assayed using the ONE-Glo™ Luciferase Assay System (Promega, Madison, WI, USA). Luciferase activities were measured using a GloMax^®^-Multi Microplate Multimode Reader (Promega Co., Sunny Vale, CA, USA).

### 3.3. Cell Proliferation Assay

Cell viabilities were evaluated using a WST (EZ-CyTox, Daeil Lab Service Co., Ltd, Seoul, South Korea). Ac2F cells (a rat liver cell line) were harvested and plated into 96-well microtiter plates at optimal seeding density (1 × 10^4^ cells per well) and preincubated in complete medium in a humidified atmosphere containing 5% CO_2_ at 37 °C for 24 h. The complete medium was removed and test substances dissolved in serum-free medium (100 μL/well) added, and cells were incubated for 6 h, 12 h, or 24 h. WST reagent (10 μL/well) was then added and incubated at 37 °C for 1 h. Absorbances were read using a iMark Microplate Absorbance Reader (Bio-Rad Laboratories, Hercules, CA, USA) at a test wavelength of 450 nm and a reference wavelength of 655 nm.

### 3.4. Molecular Docking Study

Docking calculations were performed using AutoDock Vina 1.1.2 software (The Scripps Research Institute, La Jolla, CA, USA). Default settings and the Vina scoring function were applied. For ligand preparation, Chem3D Ultra 8.0 software (CambridgeSoft Corporation, Cambridge, MA, USA) was used to convert the 2D structures of candidates into 3D structural data. Protein coordinates were downloaded from the Protein Data Bank (accession code: 2PRG). Chain A was prepared for docking within the molecular modeling software package, Chimera 1.5.3 (National Institutes of Health, Bethesda, MD, USA), by removing chain B, all ligands and water molecules (except water molecules 308, 399, 444 and 467) and by calculating protein protonation states. Polar hydrogen and setting grid box parameters were added using MGLTools 1.5.4 (The Scripps Research Institute, La Jolla, CA, USA). The analysis and visual investigation of ligand-protein interactions of docking poses were performed using PyMol v1.5 (Schrodinger LLC, New York, NY, USA).

## 4. Conclusions

In conclusion, based on the results of our pharmacophore study of the marine natural product, paecilocin A, and of synthetic PPAR-γ agonists, we designed a series of *N*-substituted phthalimides. A free hydroxyl group on the phthalimide moiety and a hydrophilic tail were found to promote PPAR-γ activation. Furthermore, docking simulation results produced some interesting correlations between 3D structures and biological activities. We believe that further intensive optimization and evaluation of this new PPAR-γ agonist scaffold are likely to be rewarding. 

## Figures and Tables

**Figure A2:**
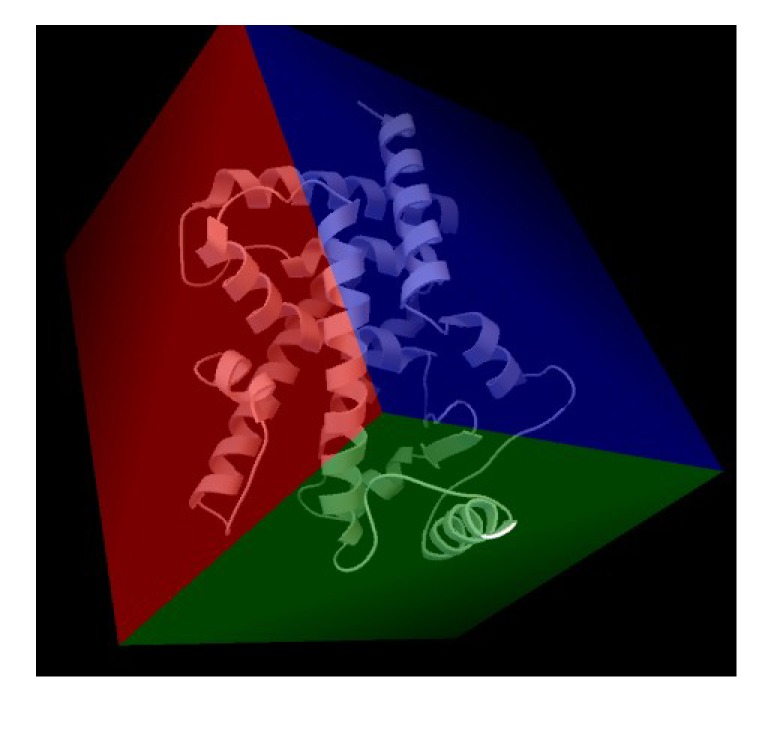
Grid box globally covering PPAR-γ protein (exhibited in MGLTools 1.5.4.).

## Appendix

Docking parameters on AutoDock Vina 1.1.2.

center_x = 49.666
center_y = −29.084
center_z = 15.217
size_x = 48
size_y = 56
size_z = 44
exhaustiveness = 100
mode = 9

**Table A1:** Docking analysis results for compounds **3**‒**14** and rosiglitazone (Rosi).

Compounds	AT ^a^	AA ^b^	NP ^c^	AI ^d^
**3**	−7.5	−6.756	4	3.50
**4**	−7.4	−6.733	4	2.35
**5**	−7.2	−6.944	0	0.00
**6**	−8.3	−7.311	3	2.33
**7**	−8.4	−7.356	4	1.75
**8**	−7.6	−6.944	4	0.75
**9**	−7.8	−7.467	4	1.00
**10**	−6.9	−6.656	3	0.67
**11**	−8.6	−7.189	4	1.00
**12**	−9.3	−8.378	4	1.11
**13**	−8.9	−8.400	4	1.67
**14**	−9.4	−8.133	3	1.67
**Rosi**	−8.2	−7.033	4	1.50

^a^ AT, affinity of the top-ranked mode; ^b^ AA, average affinity of the nine generated modes; ^c^ NP, the number of modes located in the binding pocket; ^d^ AI, the average number of H-bond interactions with key amino acid residues).
